# Alveolar leak develops by a rich-get-richer process in ventilator-induced lung injury

**DOI:** 10.1371/journal.pone.0193934

**Published:** 2018-03-28

**Authors:** Katharine L. Hamlington, Jason H. T. Bates, Gregory S. Roy, Adele J. Julianelle, Chantel Charlebois, Bela Suki, Bradford J. Smith

**Affiliations:** 1 Vermont Lung Center, Department of Medicine, University of Vermont College of Medicine, Burlington, VT, United States of America; 2 Department of Biomedical Engineering, Boston University, Boston, MA, United States of America; 3 Department of Bioengineering, University of Colorado Denver, Aurora, CO, United States of America; University of Tübingen, GERMANY

## Abstract

Acute respiratory distress syndrome (ARDS) is a life-threatening condition for which there are currently no medical therapies other than supportive care involving the application of mechanical ventilation. However, mechanical ventilation itself can worsen ARDS by damaging the alveolocapillary barrier in the lungs. This allows plasma-derived fluid and proteins to leak into the airspaces of the lung where they interfere with the functioning of pulmonary surfactant, which increases the stresses of mechanical ventilation and worsens lung injury. Once such ventilator-induced lung injury (VILI) is underway, managing ARDS and saving the patient becomes increasingly problematic. Maintaining an intact alveolar barrier thus represents a crucial management goal, but the biophysical processes that perforate this barrier remain incompletely understood. To study the dynamics of barrier perforation, we subjected initially normal mice to an injurious ventilation regimen that imposed both volutrauma (overdistension injury) and atelectrauma (injury from repetitive reopening of closed airspaces) on the lung, and observed the rate at which macromolecules of various sizes leaked into the airspaces as a function of the degree of overall injury. Computational modeling applied to our findings suggests that perforations in the alveolocapillary barrier appear and progress according to a rich-get-richer mechanism in which the likelihood of a perforation getting larger increases with the size of the perforation. We suggest that atelectrauma causes the perforations after which volutrauma expands them. This mechanism explains why atelectrauma appears to be essential to the initiation of VILI in a normal lung, and why atelectrauma and volutrauma then act synergistically once VILI is underway.

## Introduction

Acute respiratory distress syndrome (ARDS) is a life-threatening condition for which there are currently no medical therapies other than supportive care that revolves around the application of mechanical ventilation [1]. Unfortunately, mechanical ventilation comes with its own risks because of the potentially injurious stresses and strains it can inflict on an already damaged lung [2]. Such stresses and strains can cause or exacerbate the leakage of plasma-derived fluid and proteins into the airspaces of the lung where they interfere with the functioning of pulmonary surfactant [3–5], which increases surface tension at the air-liquid interface. This greatly increases the tissue stresses wrought by mechanical ventilation and thus predisposes the lung to ventilator-induced lung injury (VILI). Once VILI occurs, managing ARDS and saving the patient becomes increasingly problematic, so maintaining an intact epithelial barrier represents a crucial management goal [6].

VILI is thought to arise via two distinct mechanisms known as volutrauma and atelectrauma [7]. Volutrauma [8, 9] results when the parenchymal tissues are overstretched to the point of mechanical failure, while atelectrauma results when collapsed alveoli and airways are repetitively forced open with each breath. Volutrauma and atelectrauma represent the two key means by which the last line of defense against surfactant dysfunction, the pulmonary epithelium, becomes breached in VILI, yet their relative roles in this process remain poorly understood. In fact, we have shown that volutrauma and atelectrauma interact in a synergistic fashion [10]. The reasons for this synergy remain obscure, but they represent an important knowledge gap that needs to be addressed because they may hold the key to minimizing epithelial leak and the subsequent ravages of VILI.

Accordingly, in the present study we sought to understand how alveolocapillary barrier leak first starts, and then develops over time, as a result of injurious mechanical ventilation. We studied initially normal mice subjected to a ventilation regimen that imposed both volutrauma and atelectrauma on the lung, and observed the rate at which macromolecules of various sizes leaked into the airspaces as a function of the degree of overall injury. We used the experimental data to develop a computational model to explain the possible roles of atelectrauma and volutrauma in VILI.

## Materials and methods

### Ventilation protocol

In a protocol approved by the Institutional Animal Care and Use Committee of the University of Vermont (Protocol # 14–056), and in compliance with the Animal Welfare Act, we studied healthy 8–10 week old 16–23 g BALB/c mice (Jackson Laboratories, Bar Harbor, ME, USA). The animals were anesthetized with 120 mg/kg ketamine and 16 mg/kg xylazine via intraperitoneal (IP) injection. They were then tracheostomized and ventilated with a flexiVent small animal ventilator (SCIREQ, Montreal, QC, Canada). Continuous anesthesia was maintained with alternating doses of ketamine (60 mg/kg) and ketamine (60 mg/kg) with xylazine (8 mg/kg) at 30 min intervals administered with 0.15 ml IP 5% dextrose lactated ringers solution. Anesthesia depth was monitored via heart rate from the EKG. No animals required additional anesthetic. Pancuronium bromide (0.8 mg/kg IP) was administered at the start of ventilation to prevent spontaneous breathing efforts that interfere with the accurate measurement of lung mechanics. No signs of spontaneous breathing were subsequently observed.

The experiment began with 10 min of stabilizing ventilation at a positive end-expiratory pressure (PEEP) of 3 cmH_2_O and a tidal volume (V_T_) = 10 ml/kg. Lung function was assessed by applying a derecruitment test [11, 12] at PEEP = 0 immediately after the stabilization period. The derecruitment test began with a recruitment maneuver consisting of a 3 s ramp in airway pressure to 30 cmH_2_O followed by a 3 s breath hold. This was immediately followed by nine sequential measurements of respiratory system impedance, each obtained by applying a 2 s multi-frequency (1–20 Hz) oscillatory volume perturbation to the lungs with the flexiVent ventilator. The impedance measurements were separated by 21 s of regular mechanical ventilation (V_T_ = 10 ml/kg, PEEP = 3 cmH_2_O). Each impedance measurement was fit with the constant-phase model [13] to yield a set of 9 values of respiratory system elastance (H) that increased progressively with time at a rate reflecting the propensity of the lung to derecruit over time.

After this initial lung function assessment, the mice were divided into groups, each of which was subjected to one of four ventilation protocols. Each protocol consisted of repeated blocks that began with 310 s of mechanical ventilation with an end-inspiratory plateau pressure of 37.5 cmH_2_O. The ZEEP/Short group was ventilated with zero end expiratory pressure for approximately 30 min. The ZEEP/Mid group was ventilated with zero PEEP for approximately 60 min. The ZEEP/2xH group was ventilated with zero PEEP until H had risen to twice its baseline value. The PEEP group was ventilated with PEEP = 3 cmH_2_O for approximately 120 min as this duration was approximately equal to that of the ZEEP/2xH group and, based on our previous investigations [5, 10], does not lead to the development of obvious VILI in initially healthy mice. This experimentally applied PEEP = 3 cmH_2_O is approximately equal to the mean PEEP = 2.5 cmH_2_O reported in a recent meta-analysis [14] for standard ventilation of the non-injured lung in perioperative and intensive care unit patients. We elected not to apply the reported mean PEEP = 7.6 cmH_2_O for clinical lung-protective ventilation [14] of because of the high mortality we previously observed in mice ventilated with PEEP = 8 cmH_2_O and a plateau pressure of 35 cmH_2_O (data not shown). Details of the ventilation duration for each group are provided in Table 1. In each group, ventilation was followed immediately by a pressure-volume (PV) measurement consisting of a 3 s ramp in airway pressure to 30 cmH_2_O, a 3 s end-inspiratory hold, and then a 3 s pressure ramp down to PEEP. Finally, H was determined from two impedance measurements made as described above, separated by 12 s of ventilation at V_T_ = 10 ml/kg. Mice from the PEEP, ZEEP/Short, ZEEP/Mid, and ZEEP/≥2xH groups were then assessed for cell membrane disruption and alveolocapillary barrier permeability as described below.

**Table 1:** Lung function changes in ventilation groups.

Group	n	Ventilation Time (min)	H % Increase During Ventilation*	ΔH_0_ (cmH_2_O ml^-1^)
*Cohort 1*: *Blood-Gas Barrier Permeability*				
Control	10	––––––––No Ventilation––––––––		
PEEP3	9	122–125	10%±20% [−11%,31%]	2.3±5.2 [−3.1,7.7]
ZEEP/Short	7	30–34	2%±5% [−3%,6%]	0.3±1.1 [−0.7,7.1]
ZEEP/Mid	6	65–66	22%±18% [4%,41%]	4.5±3.3 [1.1,7.9]
ZEEP/≥2xH	9	56–151	250%±89% [175%,324%]	52.3±18.2 [37.1,67.6]
*Cohort 2*: *Cell Membrane Disruption*				
Control	5	14–17^†^	–––––	
PEEP3	5	131–132	1%±10% [−9%,11%]	0.0±2.1 [−3.4,3.4]
ZEEP/Short	5	38–40	2%±4% −1%,6%]	0.5±0.8 [−0.5,1.4]
ZEEP/Mid	5	71–76	26%±14% [14%,38%]	5.6±2.8 [2.1,9.1]
ZEEP/2xH	6	76–127	108%±16% [95%,121%]	22.7±4.2 [18.3,27.1]

*Definition of abbreviations*: PEEP, positive end-expiratory pressure; ZEEP, zero end-expiratory pressure; H, elastance. ΔH_0_ is change in H measured at PEEP = 0 cmH_2_O immediately following a recruitment maneuver at the start and end of the ventilation protocol.

*Mean ± SD, [95% CI], measurement at PEEP = 0.

^†^Control mice were protectively ventilated during the propidium iodide profusion procedure only.

### Blood-gas barrier permeability

A cohort comprised of animals from each ventilation group (n = 41) received a retro-orbital injection of fluorescent dextran conjugates (25 mg/kg each of 3 kDa Cascade Blue, 70 kDa Texas Red, and 2000 kDa Fluorescein; Thermo Fisher) five minutes before the end of ventilation. The PEEP3 group had one set of PV and impedance measurements made at PEEP = 0 at the end of the protocol for comparison to final measurements in the ZEEP groups. After removal from the ventilator, 1 ml PBS was instilled through the tracheal cannula and suctioned back to BALF. Blood was collected via cardiac puncture. Control animals were not ventilated and received the retro-orbital injection five minutes after tracheostomy followed by BALF and blood collection five minutes later. BALF and blood were centrifuged at 1600 rpm and 2400 rpm, respectively, for 10 min, and the supernatant was stored at −80°C. Black 96-well plates were filled with four undiluted 100 μL replicates of BALF and two 100 μL replicates of serum (1:4 dilution in PBS). Fluorescence of the dextran conjugates in the samples was determined using a BioTek Synergy HTX plate reader (Winooski, VT, USA).

### Cell membrane disruption

Following the final PV and impedance measurements at PEEP = 0, a second cohort of mice (n = 26) were perfused at 20 cmH_2_O with 5 ml of 25 μg/ml propidium iodide (PI) [15] in heparinized saline through the right ventricle for five minutes while ventilation continued. PI is membrane impermeant and is excluded from live and intact cells. If the cell membrane is disrupted, PI binds to nucleic acids and the fluorescence intensity increases 20–30 fold. In addition to cells directly damaged by mechanical ventilation, PI will label macrophages undergoing apoptosis and neutrophils presenting neutrophil extracellular traps (NETs) as part of the inflammatory response to VILI [16]. Control mice had one derecruitment test performed at PEEP = 0 and were protectively ventilated at V_T_ = 10 ml/kg with PEEP = 3 cmH_2_O during the PI perfusion. After removal from the ventilator, 1 ml 1:1 Tissue Tek OCT and PBS was instilled into the lungs and the trachea was ligated. The lungs were then perfusion fixed with 4% paraformaldehyde at 20 cmH_2_O for five minutes after which they were excised and immersion fixed for 24 hours at 4°C. Lung volume was determined by volume displacement and then the lungs were snap-frozen in OCT and stored at −80°C. For the stereology analysis, 25 μm lung sections were selected via systematic uniform random sampling (SURS) [17], and cell nuclei were stained with 1:500 DAPI (Life Technologies, Carlsbad, CA) in 1%BSA+PBS. SURS optical disector pairs (3 μm z-distance) were imaged at 40x with a Zeiss LSM 510 META confocal microscope (Carl Zeiss Microimaging, Thornwood, NY) to estimate the total number of membrane-disrupted (PI+) and DAPI+ cells in the lungs [18].

### Statistical analysis

Statistical significance was accepted at p<0.05. Welch’s one-way ANOVA with Games-Howell post hoc comparison was used to compare differences between groups when the assumption of homogeneity of variance was violated; one-way ANOVA with Tukey HSD post hoc test was used otherwise. Kruskal-Wallis H test was used when the Shapiro-Wilk test for normality failed. Analysis was completed in Matlab (MathWorks, Natick, MA) and SPSS (IBM, Armonk, NY). All values are mean±SD [95% CI] unless indicated otherwise.

## Results

### Blood-gas barrier permeability

**Fig 1:**
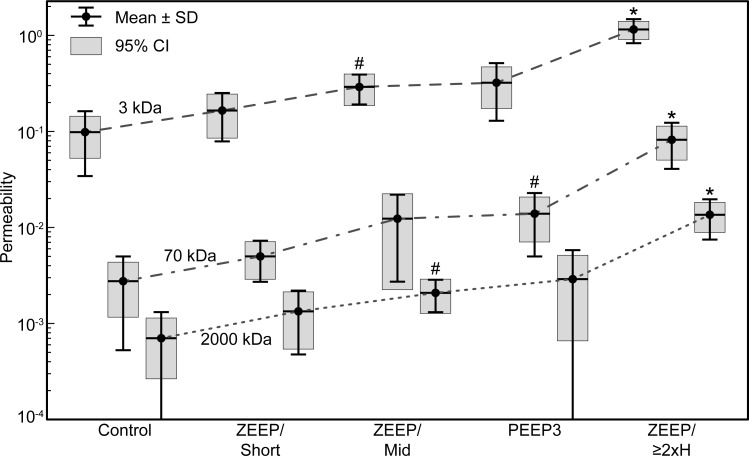
Blood-gas barrier permeability by ventilation group and dextran conjugate size. Permeability is defined as the ratio of bronchoalveolar lavage fluid fluorescence to serum fluorescence for each size of dextran conjugate (3 kDa, 70 kDa, and 2000 kDa). *Significant increase in permeability from all other groups within dextran size. ^#^Significant increase from Control (p<0.05). ZEEP: zero end-expiratory pressure, PEEP3: positive end-expiratory pressure = 3 cmH_2_O, Short: ventilation time 30 min, Mid: ventilation time 60 min, ≥2xH: ventilation until elastance (H) at least doubled.

A retro-orbital intravenous injection of 3, 70, and 2000 kDa fluorescent dextran conjugates was administered to a cohort of mice five minutes before the end of mechanical ventilation, and blood-gas barrier permeability μ was quantified as the bronchoalveolar lavage fluid (BALF) fluorescence relative to serum fluorescence [19]. Replicates from microplate readings were averaged (coefficient of variation = 2.7% ± 2.3%, range 0.03% -13.7%). As determined by Welch’s one-way ANOVA, barrier permeability was significantly different between ventilation groups for the 3 kDa (p = 1.25 × 10^−6^), 70 kDa (p = 1.58 × 10^−4^), and 2000 kDa (p = 9.43 × 10^−5^) dextran conjugates. The permeability to the three conjugate sizes, μ_3_, μ_70_, and μ_2000_, was significantly greater for ZEEP/≥2xH than for all other groups (Games-Howell post hoc, p ≤ 6.60 × 10^−3^). Additionally, μ_3_ and μ_2000_ were greater in the ZEEP/Mid group than in the Control group (p = 2.03 × 10^−2^ and p = 3.00 × 10^−2^), and μ_70_ was greater in the PEEP group than in the Control group (p = 3.33 × 10^−2^). The permeability results are shown in Fig 1.

Within all groups, the magnitude of μ_3_ was significantly greater than μ_70_, which was significantly greater than μ_2000_ (pair-wise comparisons with Bonferroni adjustment, p ≤ 0.023), with the exception that there was no significant difference between μ_70_ and μ_2000_ permeability in the ZEEP/Mid group. However, the proportions of μ_3_ to μ_2000_ and μ_70_ to μ_2000_ permeability were not significantly different between groups (Kruskal-Wallis H test, p = 0.129 and 0.375, respectively). Over all mice, the median μ_3/2000_ ratio was 123.39 (IQR 88.12−179.48), and the median μ_70/2000_ ratio was 5.34 (IQR 3.64−6.66). This indicates that any injury to the blood-gas barrier that increased the permeability to 3 kDa- and 70 kDa-sized particles did so in proportion to μ_2000_, but at rates 120 and 5 times greater, respectively. Robust least squares linear regression with bisquare weights across all groups confirmed a strong linear relationship between μ_3_ and μ_2000_ (μ_3_ = 76.59μ_2000_+ 0.084, r^2^ = 0.947) and between μ_70_ and μ_2000_ (μ_70_ = 5.69μ_2000_−6.78 × 10^−4^, r^2^ = 0.981).

In contrast to the consistent μ_3/2000_ and μ_70/2000_ relationships, the proportion of μ_3_ to μ_70_ was significantly different between groups (Kruskal-Wallis H test, p = 0.006). From pairwise comparison with Bonferroni correction, this ratio was significantly smaller for the ZEEP/≥2xH group (mean rank = 8.33) than the ZEEP/Short group (mean rank = 28.71, p = 0.007) and Control group (mean rank = 25.40, p = 0.019). The μ_3/70_ ratios for the ZEEP/Mid (mean rank = 23.87) and PEEP3 (mean rank = 21.00) groups were not statistically different from the ZEEP/≥2xH group. Thus, the blood-gas barrier was generally more permeable to 70 kDa particles relative to 3 kDa particles in the most injured group (ZEEP/≥2xH median μ_3/70_ = 13.88, IQR 12.74–18.30) compared to the other groups (combined median μ_3/70_ = 24.47, IQR 20.88–40.11). The relationship between μ_3_ and μ_70_ among all mice was also strongly linear (μ_3_ = 12.92μ_70_ + 0.099, r^2^ = 0.949) with a slope similar to the median μ_3/70_ ratio of the ZEEP/≥2xH group. One observation from the linear regression analysis worth noting is that the blood-gas barrier was permeable to 3 kDa dextran but not to 70 kDa or 2000 kDa dextran when there was little or no injury due to ventilation (y-intercept > 0).

### Cell membrane disruption

**Fig 2:**
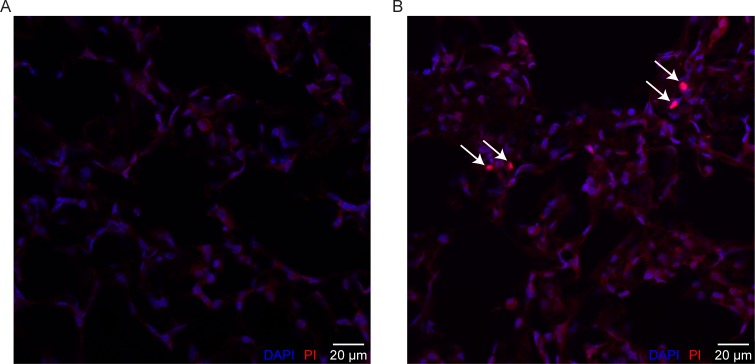
Propidium iodide (PI) as indicator of lung cell injury. Representative images from the Control (A) and ZEEP/≥2xH group (B) groups showing PI+ nuclei (red cells, white arrows). Fluorescence intensity increases ~20-fold when PI binds to nucleic acids. ZEEP/≥2xH group was ventilated with zero end-expiratory pressure until elastance at least doubled; control group was not ventilated.

**Fig 3:**
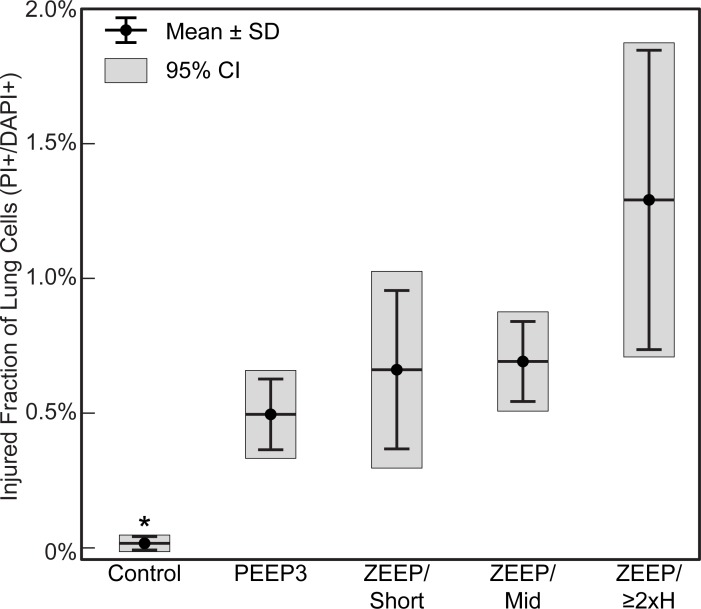
Injured fraction of lung cells (PI+/DAPI+) by ventilation group. *Significantly less than all groups (p≤0.036). PI: propidium iodide, marker of cell membrane disruption, ZEEP: zero end-expiratory pressure, PEEP3: positive end-expiratory pressure = 3 cmH_2_O, Short: ventilation time 30 min, Mid: ventilation time 60 min, ≥2xH: ventilation until elastance (H) at least doubled.

A second cohort of mice was perfused with PI to demarcate cell membrane disruption. Fig 2B shows a representative image of a lung slice from the ZEEP/≥2xH group with PI+ nuclei indicating cell membrane disruption (red cells, white arrows). A representative image from the control group is shown in Fig 2A. There were 3.21 ± 0.55 [2.99, 3.43] x 10^8^ DAPI+ cells in the mouse lungs (n = 26 in cohort, no differences in lung cell count between groups). Fig 3 shows the injured fraction of lung cells (PI+/DAPI+). The number of PI+ cells and the injured fraction were significantly smaller in the Control group than all other groups (Welch’s ANOVA with Games-Howell post-hoc, p ≤ 0.046) and were not significantly different from zero in the Control group only (5.89 ± 8.43 [−4.57, 16.35] x 10^4^ PI+ cells). Although greater than zero in all other groups, there were no significant differences in number of PI+ cells or injured fraction between the ventilated groups due to variability among mice.

### Lung function degradation

**Fig 4:**
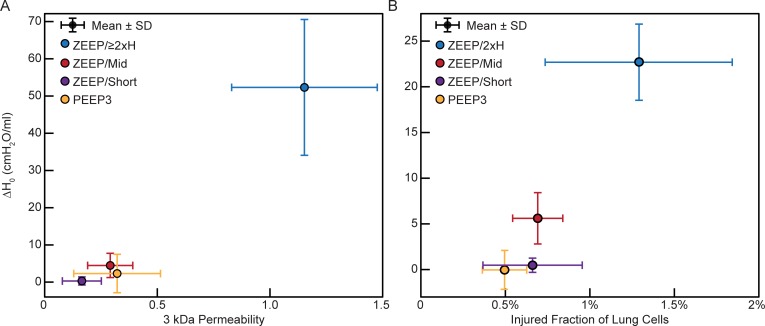
Lung function. Change in elastance measured at PEEP = 0 between the start and end of ventilation (ΔH_0_) versus (A) permeability of 3 kDa dextran (BALF/Blood fluorescence, cohort 1 mice) and (B) fraction of injured lung cells (PI+/DAPI+, cohort 2 mice). PI: propidium iodide, marker of cell membrane disruption, ZEEP: zero end-expiratory pressure, PEEP3: positive end-expiratory pressure = 3 cmH_2_O, Short: ventilation time 30 min, Mid: ventilation time 60 min, ≥2xH: ventilation until elastance (H) at least doubled.

Permeability and cell membrane disruption were compared to the change in H measured at PEEP = 0 cmH_2_O immediately following a recruitment maneuver at the start and end of the ventilation protocol (ΔH_0_). This is shown in Fig 4A for the 3 kDa dextran (cohort 1) and Fig 4B for the injured fraction of lung cells (cohort 2); the trends were similar for the 70 kDa and 2000 kDa dextrans (data not shown). Leak, cell injury, and ΔH_0_ all increased from the ZEEP/Short group to the ZEEP/Mid group to the ZEEP/≥2xH group. Lung function changes are listed in Table 1. Increase in H indicates increased lung stiffness; ΔH_0_ was greater in the ZEEP/≥2xH group than all other groups in their respective cohorts (p ≤ 5.09 x 10^−4^). H_0_ also increased over the course of ventilation in the ZEEP/Mid groups, but H_0_ did not significantly change in the ZEEP/Short groups or either PEEP3 group.

### Size distribution of alveolar leaks

We used our measurements of the relative permeabilities of the blood-gas barrier to 3, 70, and 2000 kDa dextran conjugates to estimate the distribution of hole sizes through which material escapes from the blood and interstitial space into the airspace. Let *A*_2000_ be the area of a (circular) hole just big enough to allow passage of a 2000 kDa dextran molecule. The 2000 kDa dextran molecules then pass into the airspaces through holes of area *A*_2000_ or bigger. We assume that the total rate at which dextran molecules pass through all these holes is proportional to μ_2000_. Precisely how this rate depends on hole size is not immediately obvious, but the following considerations suggest a range of possibilities. One possibility is that dextran molecules are carried through a hole as passive passengers on a flow of plasma-derived fluid and protein. If the holes contribute little to the resistance that controls the flow, then flow will be proportional simply to hole area. On the other hand, if the holes make a substantial contribution to resistance then one would expect flow to be proportional roughly to the square of hole area (e.g., as in laminar Poiseuille flow). Alternatively, rather than being passive passengers, the dextran molecules might interact with the holes in some way. For example, the flow of particles through apertures has been modeled as depending on area to the ¾ power [20]. Therefore, it seems reasonable to suppose that flow of dextran molecules through a hole of area *A* is proportional to *A*^*α*^, where *α* is between 0.75 and 2.

The total flow of the 2000 kDa molecules, *Q*_2000_, which is reflected in μ_2000_, will thus occur at a rate that is a function of the cumulative area of the holes of area *A*_2000_ or above:

$$Q_{2000}=k\;\int_{A_{2000}}^{\infty }A^{\alpha }f(A)dA,$$ (1)

where *f*(*A*) is a hole size density function such that *f*(*A*)*dA* is the number of holes having areas between *A* and *A* + *dA*, and *k* is a constant of proportionality with units of flow if we normalize *A* to the area of a hole that allows passage of 1 kDa dextran molecules and larger. Similarly,

$$Q_{70}=k\;\int_{A_{70}}^{\infty }A^{\alpha }f(A)dA=Q_{2000}+\int_{A_{70}}^{A_{2000}}A^{\alpha }f(A)dA$$ (2)

and

$$Q_{3}=Q_{70}+\int_{A_{3}}^{A_{70}}A^{\alpha }f(A)dA.$$ (3)

We fit these expressions to the mean values of permeability measured in each experimental group of mice by minimizing the objective function

$${J=(\rho \mu_{2000}-Q_{2000})}^{2}+{\left(\rho \mu_{70}-Q_{70}\right)}^{2}+{\left(\rho \mu_{3}-Q_{3}\right)}^{2},$$ (4)

where *ρ* is a constant of proportionality with units of flow divided by permeability. The objective function (*J*) was selected to equally penalize differences between the predicted and measured barrier permeability to the three sizes of dextran used in the experiment. We used the reported values 3 nm, 13 nm, and 54 nm for the hydrodynamic diameter of 3 kDa, 70 kDa, and 2000 kDa dextran, respectively, to compute area [21, 22].

**Fig 5:**
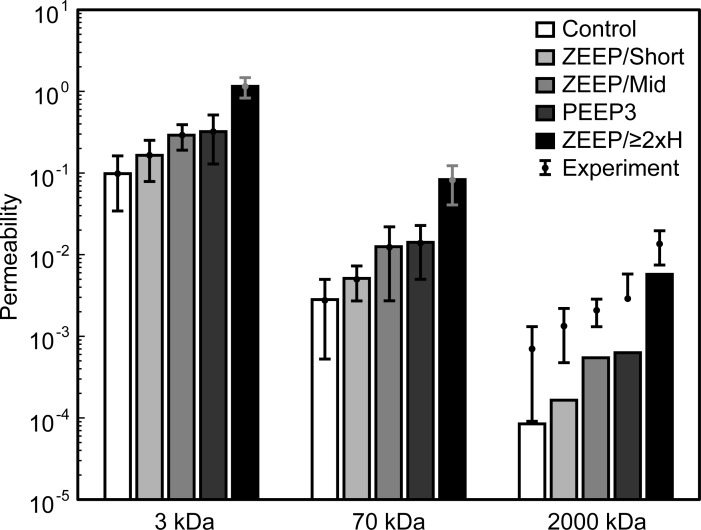
Measured and predicted barrier permeability. Comparison between the predicted (bars, *α* = 1.0) and measured (points) blood-gas barrier permeability to 3kDa, 70 kDa, and 2000 kDa dextrans. Error bars show the standard deviation of the experimental measurements.

Minimizing *J* in Eq 4 required that we assume some functional form for the density function *f*(*A*). We found by trial and error that a power-law of the form

$$f(A)=f_{0}A^{\beta }$$ (5)

provided good fits to the data in each group, where *f*_0_ and *β* are constants. We fit the model using 13 different values of α (Eqs 1–3) ranging from 0.75 to 2.0 and found that the best-fit values of *f*_0_ were independent of *α* (Table 2). By contrast, the predicted values of *β* in each group were linearly related to *α* with a slope of -1.0 and R^2^ = 1.0 (Table 2). *β* ranged from -2.64 (ZEEP/≥2xH group, *α* = 0.75) to *β* = -4.21 (Control group, *α* = 2.0). It is evident that *f*_0_ (the y-intercept of the log-log plot) decreases from the ZEEP/≥2xH group to the Control group, with little difference between the PEEP and ZEEP/Mid groups. Conversely, the rate at which the number of holes decreases with increasing diameter (*β*) increases from the ZEEP/≥2xH group to the Control group, with little difference between the PEEP and ZEEP/Mid groups or between the ZEEP/Short and Control groups. The errors between the predicted and measured permeabilities were 0.001%–0.005% for 3 kDa, 1.3%–2.6% (*Q* > $\bar{\mu }$) for 70 kDa, and 57%–88% (*Q* < $\bar{\mu }$) for 2000 kDa, and these errors were independent of *α*. Fig 5 the shows the predicted (bars, *α* = 1.0) and measured (points) blood-gas barrier permeability to 3, 70, and 2000 kDa dextrans. These predictions are representative of the results for 0.75 ≤ *α* ≤ 2.0 because there was less than 10^−12^% variation between the predicted permeabilities at different *α*. This analysis suggests that damaging ventilation (ZEEP/≥2xH) increases both the overall number of perforations in the blood-gas barrier as well as the sizes of these perforations, and that these predictions are equally applicable to flows described by 0.75 ≤ *α* ≤ 2.0 and are independent of the physical mechanisms involved in transport of particles across the epithelial barrier.

**Table 2:** Permeability model best-fit parameters.

Group	n	*f*_0_	*β*
Control	10	1.27	-(2.21 + α)
PEEP3	9	2.74	-(2.06 + α)
ZEEP/Short	7	1.98	-(2.18 + α)
ZEEP/Mid	6	2.53	-(2.07 + α)
ZEEP/≥2xH	9	5.92	-(1.89 + α)

Best-fit values of the parameters *f*_0_ and *β* in Eq 5. *β* decreased linearly with increasing α and the root mean square error (RMSE) < 10^−4^. *f*_0_ was independent of α with the RMSE < 10^−3^.

### Modeling alveolar leak progression by a rich-get-richer mechanism

Power-law processes abound in nature for reasons that have engendered many theories [23, 24]. One of the more successful of these is based on the rich-get-richer mechanism that potentially explains, for example, why the number of connections to sites on the internet from other sites exhibits a power-law distribution [25]. The rich-get-richer mechanism suggests itself as an explanation for the power-law form of Eq 5 and thus for the underlying process by which injurious mechanical ventilation causes VILI to develop, as the following considerations illustrate. The alveolar epithelial lining normally presents an intact barrier that prevents fluid and protein in the alveolar interstitial space from exiting into the airspaces. The serious physiological manifestations of VILI begin to occur when this barrier is breached by the stresses of injurious mechanical ventilation. If injurious ventilation persists, this injury will worsen both through the generation of new epithelial perforations as well as the widening of those perforations already in existence. Furthermore, it seems reasonable to suppose that perforations will occur preferentially in those regions of the epithelial lining where, for whatever reason, injurious stresses are highest and/or mechanical strength is lowest. For the same reasons, one would also expect that the perforations in these regions would tend to expand the most quickly.

We can model this rich-get-richer mechanism in very simple terms using the well-known preferential attachment algorithm [26]. The model starts with a single epithelial hole of area 1 (in arbitrary units). At each step in the simulation we draw a random number *x* from a uniform distribution on the interval [0, 1]. If *x* < *r*, for some 0 < *r* < 1, then another hole of area 1 is created. If *x* ≥ *r* then the area of the existing hole is increased by 1. This process is repeated *m* times with the additional condition that when *x* ≥ *r* and there are 2 or more holes, the area of only a single hole is increased by 1, with the probability of a particular hole being chosen for widening being proportional to its current area. The theoretical distribution, $\hat{f}(A)$, of hole area given by this process is proportional to $A^{-\hat{\beta }}$, where

$$\hat{\beta }=1+\frac{1}{r}.$$ (6)

**Fig 6:**
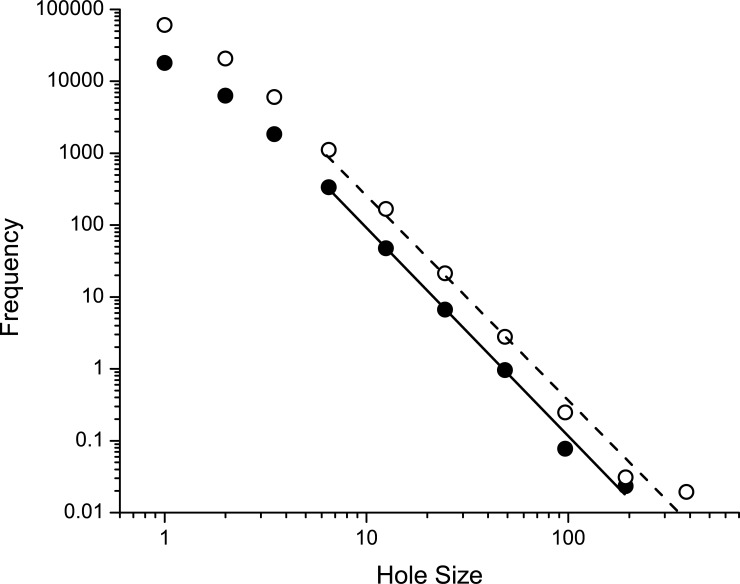
Rich-get-richer model predictions. Histogram of hole diameters for the rich-get-richer simulation run until *m* = 30,000 (closed symbols) and *m* = 100,000 holes (open symbols). Straight lines were fit to each set of points after discarding the first 3 in each case. The slopes of the relationships beyond the first 3 points are -2.9 (SE 0.1) for *m* = 30,000 and -2.9 (SE 0.2) for *m* = 100,000.

Fig 6 shows $\hat{f}(A)$ obtained with *m* = 30,000 and 100,000, with *r* = 0.5. After an initial transient, the slopes of the logarithm of $\hat{f}(A)$ versus the logarithm of *A* are very similar to the theoretical value of -3, and are also within the range of values of *β* found experimentally (Table 2).

## Discussion

It is well known that VILI leads to perforations in the normally intact barrier presented by the pulmonary endothelium and epithelium, and that leakage of plasma-derived material through such perforations leads to the main manifestations of VILI on lung mechanics that are caused by deactivation of pulmonary surfactant [3–5]. It is therefore no surprise that we found increasingly injurious modes of mechanical ventilation to cause increasing barrier permeability (Fig 1 and Table 1). What our study shows for the first time, however, is that VILI causes alveolocapillary barrier leaks to appear with a power-law distribution of sizes (Eqs 1–3, Table 2, Fig 5). Furthermore, the exponent of the power-law remained relatively unchanged, for a given value of *α*, even as the total leak increased by orders of magnitude. These observations can be explained by a rich-get-richer mechanism in which epithelial perforations are both created and amplified with relative probabilities that are roughly equal and that do not change as injury progresses (Fig 6).

On the one hand, the appearance of a power-law in this context has a familiar ring to it; power-laws crop up all over the natural world in numerous guises, and indeed seem to be a signature of complex dynamic systems in general [23–25]. On the other hand, the power-law feature of epithelial perforations in VILI, and the rich-get-richer mechanism that potentially affords a mechanistic explanation for this observation, may shed light on a previous experimental observation from our laboratory that has thus far resisted satisfactory explanation. We have strong evidence in mice that volutrauma alone does not instigate VILI in a normal lung over experimentally tractable timescales; excessive tidal volume is either borne without apparent difficulty, or else the lung experiences a sudden catastrophic pneumothorax [5, 10, 27, 28]. Progressive VILI appears to require the simultaneous presence of both volutrauma and atelectrauma. Indeed, avoidance of atelectrauma seems to be key to avoiding the initiation of VILI even in the presence of very high inflation pressures in both septic pigs and in patients at risk for developing ARDS [29–31]. The modeling results of the present study indicate that VILI develops through the stochastic appearance of new epithelial perforations roughly 50% of the time (0 < *r* < 0.5) and the widening of an existing hole the remaining 50% of the time (0.5 ≤ *r* < 1). Since a new hole must presumably be started before it can be subsequently widened, we might speculate that atelectrauma is primarily responsible for the production of new perforations through its direct damaging effects on epithelial cells [32, 33], while volutrauma acts to enlarge existing holes via the stretch that it imposes on the alveolar tissues [34]. This theory may partially explain why recruitment maneuvers and PEEP titration were associated with increased all-cause ARDS mortality in a recent randomized trial [35]. Likewise, increased driving pressure was associated with increased ARDS mortality in a retrospective analysis [36]. In both cases, elevated pressures were applied to lungs with existing injury and our model suggest this could result in enlargement of alveolocapillary perforations.

It is important to consider that other factors beyond the mechanical forces of volutrauma and atelectrauma can contribute to the genesis and expansion of alveolocapillary barrier disruptions. For example, neutrophil migration from the alveolar capillaries into the airspace might result in increased paracellular permeability [37] due to the mechanical forces that arise as the neutrophils squeeze between adjacent epithelial cells [38–40], thus contributing to the generation of new perforations that are later subject to expansion as described in our simulations. Neutrophils also release a cocktail of proteases, cationic peptides, and reactive oxygen species that can degrade the integrity of the alveolocapillary barrier [41]. These chemical stimuli could cause new perforations and facilitate the expansion of existing disruptions by damaging junctional proteins and triggering apoptosis [42].

Neutrophil-induced generation of new perforations in the alveolocapillary barrier may help explain the significant increase in 70 kDa dextran permeability and PI+ cells in the PEEP3 group. The level of injury in that group was less severe than in animals ventilated at PEEP = 0 with equivalent inspiratory pressures and durations, which we attribute to reduced atelectrauma. However, it is possible that the addition of 3 cmH_2_O PEEP was sufficient to completely prevent atelectrauma. If that is the case, then high pressure ventilation with PEEP may represent a different mechanism of leak initiation and enlargement than in the ZEEP cases. Nevertheless, the distribution of hole sizes in both ZEEP and PEEP ventilation are well described by the power law model.

Another aspect of VILI that is crucial for patient survival is the extent to which perforations in the epithelium can be repaired, and the rapidity with which this happens. A significant fraction of patients with ARDS do survive [43], and indeed their survival relies exclusively on the body’s ability to recover spontaneously. Direct evidence that damaged epithelial cells can repair themselves in acute lung injury has also been obtained in the animal laboratory [15]. The dynamics of this repair have yet to be elucidated in detail, but one might imagine that small perforations in the alveolar epithelial membrane could reanneal relatively quickly, while larger levels of injury that cause damage to cell-cell junctions, or even cell death, could take longer to repair. The ultimate fate of the ventilated ARDS lung would then depend on whether the repair processes are out-competed by those that cause damage. Being able to model these competing processes could guide the use of mechanical ventilation in ARDS patients so as to favor repair over damage, and thus increase the likelihood of survival.

Understanding the dynamics of VILI also requires consideration of when alveolar leak and its mechanical consequences begin to manifest relative to the beginning of actual physical injury to the alveolocapillary barrier. These events are not necessarily contemporaneous, as comparison of Fig 4A and 4B shows. There is a suggestion in these figures that cell injury as measured by PI incorporation (Figs 2 and 3) precedes leak in the early stages of injury, perhaps because when tears in the plasma membrane are small they may still be repairable and do not affect the cell’s ability to maintain an effective barrier. Once the membrane disruptions reach a certain size, however, one would expect the mechanical integrity of the cell, and possibly even its survivability, to be compromised. This would then allow the leak-exacerbating effects of volutrauma to come into play. In any case, it appears from our data that few cells need to be damaged before a physiologically significant leak takes hold (Fig 3). For example, there were roughly 4 x 10^6^ PI+ cells in the ZEEP/≥2xH group. This represents only a small fraction of all cells counted in the lung (~1.3%). However, this small percentage of injured cells could have a marked impact because the total number of injured cells is approximately twice the 2.3 x 10^6^ alveoli in the mouse lung [44]. Furthermore, as explained above, not all these PI+ cells contribute to leak, presumably because their levels of injury were not great enough to allow transit of material across the blood-gas barrier. A fraction of the PI+ cells might be NETs or macrophages undergoing apoptosis, further reducing the number of injured alveolar epithelial and endothelial cells contributing directly to leak. Indeed, we have previously shown that physical damage to the epithelium is visible via scanning electron microscopy after lungs have been subjected to a level of injurious ventilation similar to that of the ZEEP/≥2xH group, but such damage is not apparent after ventilation similar to the less injured groups of the present study [45]. Interestingly, our predictions indicate that control animals also have a small number of alveolocapillary holes (Fig 5), so perhaps there is a baseline level of epithelial permeability that does not compromise the structural integrity of the barrier.

Our study thus presents some intriguing data indicative of a power-law distribution of epithelial hole sizes. We suggest that this can be explained by a rich-get-richer mechanism in which atelectrauma causes holes to form while volutrauma causes the holes to expand. Nevertheless, these findings must be viewed relative to a number of study limitations. First of all, it is not entirely clear how to model the rate at which dextran molecules flow through epithelial holes as a function of hole size. We presented arguments that this rate should be proportional to *A*^*α*^, where 0.75 < *α* < 2, but where *α* should actually be placed within this range remains unknown. We also did not take into account in our model the possibility that two nearby holes might coalesce to produce a single larger hole. Such a process would likely still give rise to a power law, although possibly with a somewhat different exponent. Another potential limitation of our study is that we generated VILI in initially normal mice simply by ventilating them in highly injurious manners. This has no direct correlate in the clinic where VILI may accompany already existent ARDS even when mechanical ventilation is applied in a careful manner. We therefore do not know if clinical VILI would also manifest as a power-law distribution of hole sizes that progress as in Fig 5. We also studied the development of VILI over a relatively short time period of only a few hours, whereas clinical VILI may manifest over days. This longer time-scale could allow for a greater influence of the dynamics of reparative processes on the evolution of epithelial leak. In addition, there are obvious differences between mice and humans that could have an important bearing on our results, probably the most obvious being the negligible effect of the gravitational gradient on ventilation and perfusion in the tiny mouse lung relative to the much larger human organ. These limitations aside, however, we can at least begin to develop a mechanistic theory about how alveolar leak develops with VILI, and possibly what the relative roles of volutrauma and atelectrauma are in this development.

## Conclusions

The processes leading to compromise of the alveolar epithelium in VILI give rise to a power-law distribution of blood-gas barrier perforation sizes. Such a distribution can be recapitulated computationally using a rich-get-richer scheme in which atelectrauma causes, with roughly equal probability, either the formation of a new small perforation or the expansion of an existing perforation, with larger perforations being more likely to become expanded than smaller ones. This potentially explains why atelectrauma appears to be necessary to get VILI started in an initially normal lung, and how volutrauma then builds on this injury. We suggest this may provide a starting point for devising quantitative approaches to VILI minimization.
